# Supplementary material for: Impact of Sirolimus versus Mycophenolate Mofetil on Kidney Function after Calcineurin Inhibitor Dose Reduction in Liver Transplant Recipients
Source: Pharmaceuticals (Basel). 2023 Jul 31;16(8):1087. doi: 10.3390/ph16081087 (PMC10457954; doi:10.3390/ph16081087)
Supplement: Supplementary file 1 [file pharmaceuticals-16-01087-s001.zip › pharmaceuticals-2481109-supplementary.pdf]

Supplemental Table S1. Comparative risk of adverse kidney outcomes between sirolimus and MMF.

	AKD			EGFR decline > 30% from baseline		
	**aHR**	**95% CI**	***p-value***	**aHR**	**95% CI**	***p-value***
**Sirolimus vs MMF**	1.04	(0.70–1.55)	0.83	2.09	(1.33–3.28)	0.001
Baseline eGFR, mL/min/1.73 m^2						
<90	1.00	reference		1.00	reference	
≧90	1.71	(1.02–2.89)	0.04	2.52	(1.42–4.49)	0.002
**Baseline characteristic**						
Male vs female	0.86	(0.54–1.39)	0.55	0.89	(0.54–1.46)	0.64
Age, years	1.03	(1.01–1.06)	<0.01	1.05	(1.02–1.07)	0.001
Alcohol	1.14	(0.64–2.04)	0.65	0.61	(0.31–1.22)	0.16
BMI	0.99	(0.95–1.04)	0.72	0.97	(0.92–1.02)	0.26
MELD score	1.03	(1.00–1.06)	0.03	1.03	(1.00–1.06)	0.06
Hypertension	1.10	(0.60–1.99)	0.76	1.04	(0.55–1.96)	0.91
DM	1.29	(0.78–2.15)	0.32	1.01	(0.59–1.74)	0.97
HBV	0.80	(0.47–1.34)	0.39	1.17	(0.66–2.07)	0.59
HCV	0.92	(0.53–1.60)	0.77	0.84	(0.46–1.54)	0.57
HCC	1.03	(0.61–1.76)	0.90	1.07	(0.61–1.88)	0.81
**Labs**						
Baseline albumin, g/dl	-	-	-	0.77	(0.61–1.88)	0.81
Baseline Hb, g/dl	1.03	(0.66–1.60)	0.91	0.93	(0.83–1.04)	0.19
Proteinuria> 1+	1.13	(0.54–2.36)	0.75	0.84	(0.37–1.91)	0.68
Tacrolimus trough level (index date)	1.09	(0.98–1.22)	0.12	0.92	(0.81–1.04)	0.17
Tacrolimus trough level (6 month)				1.02	(0.93–1.12)	0.67
**prior medications**						
ACEI/ARB	0.71	(0.39–1.30)	0.27	1.44	(0.81–2.55)	0.21
Statins	1.34	(0.57–3.17)	0.50	2.84	(1.35–5.99)	0.006
Diuretic	2.69	(0.31–23.22)	0.37	1.26	(0.15–10.7)	0.83
NSAIDs	0.79	(0.1– 6.09)	0.82	2.54	(0.54–11.9)	0.24

ACEI: angiotensin-converting enzyme inhibitor; aHR: adjusted hazard ratio; AKD: acute kidney disease; ARB: angiotensin receptor blocker; BMI: body mass index; CI: confidence interval; DM: diabetes mellitus; eGFR: estimated glomerular filtration rate; HBV: hepatitis B virus; HCV: hepatitis C virus; HCC: hepatocellular carcinoma; MELD: model for End-Stage liver disease; MMF: mycophenolate mofetil; NSAID: nonsteroidal anti-inflammatory drugs.
